# Sex-specific mediating effect of gestational weight gain between pre-pregnancy body mass index and gestational diabetes mellitus

**DOI:** 10.1038/s41387-022-00203-5

**Published:** 2022-04-25

**Authors:** Shuang Zhang, Jingyu Wang, Fang Xu, Juhong Yang, Yongzhang Qin, Junhong Leng, Nan Li, Jia Guo, Xiaochen Li, Zhong’ai Gao, Xiaofang Shen, Hui Gao, Baocheng Chang, Hong Zhu

**Affiliations:** 1 NHC Key Laboratory of Hormones and Development, Tianjin Key Laboratory of Metabolic Diseases, Chu Hsien-I Memorial Hospital & Tianjin Institute of Endocrinology, Tianjin Medical University, Tianjin, China; 2 Tianjin Women’s and Children’s Health Center, Tianjin, China; 3 Department of Endocrinology, Inner Mongolia Baogang Hospital, Inner Mongolia Autonomous Region, Baotou, China; 4 Department of Endocrinology, First Affiliated Hospital of Gannan Medical University, Ganzhou, Jiangxi China; 5 Department of Obstetrics and Gynaecology, Beijing Tiantan Hospital, Capital Medical University, Beijing, China; 6 Department of Epidemiology and Biostatistics, School of Public Health, Tianjin Key Laboratory of Environment, Nutrition and Public Health, Tianjin Medical University, Tianjin, China

**Keywords:** Gestational diabetes, Weight management, Lifestyle modification, Risk factors, Epidemiology

## Abstract

**Background:**

Inappropriate weight gain may increase the risk of gestational diabetes mellitus (GDM). However, the relationship between pre-pregnancy body mass index (BMI), weight gain, and GDM has not been precisely quantified. This study aimed to explore whether gestational weight gain played a mediating role between pre-pregnancy BMI and GDM and whether the mediating effect was sex specific.

**Methods:**

This study established a population-based observational cohort to assess weight gain in pregnant women. Mediation analyses were performed to quantify whether weight gain mediated the association between pre-pregnancy BMI and GDM.

**Results:**

A total of 67,777 pregnant women were included in the final analysis, among whom 6751 (10.0%) were diagnosed with GDM. We verified that both pre-pregnancy BMI and weight gain were associated with GDM, and that BMI negatively contributed to weight gain. We also found that weight gain had a significant mediating effect on the relationship between pre-pregnancy BMI and GDM (*Z*_*a*_ *×* *Z*_*b*_ confidence intervals [CIs] 0.00234–0.00618). Furthermore, the effect was sex-specific, in that it was only significant in overweight women carrying female fetuses (*Z*_*a*_ *×* *Z*_*b*_ CIs 0.00422–0.01977), but not male fetuses (*Z*_*a*_ *×* *Z*_*b*_ CIs −0.00085 to 0.01236).

**Conclusions:**

Weight gain during pregnancy had a fetal sex-specific mediating effect between pre-pregnancy BMI and GDM.

## Introduction

Gestational diabetes mellitus (GDM) is one of the most common endocrine and metabolic diseases, characterized by temporary hyperglycemia or glucose intolerance with onset or first recognition during pregnancy [1, 2]. It impairs the health of mothers and offspring and creates huge social, economic, and health burdens at both the population and individual levels [3, 4]. Although the prevalence of GDM varies widely due to demographic and diagnostic criteria differences [5], it continues to increase worldwide. In addition, maternal obesity and Asian ethnicity are established risk factors for GDM. A meta-analysis from East Asia and Southeast Asia showed that the prevalence of GDM in China was reported to be 11.91%, which was much higher than that of Japan, Korea, and Thailand [6]. In the past decade, many studies have focused on screening high-risk groups of and lifestyle interventions for GDM [7–9].

Furthermore, gestational weight gain (GWG) is an important part of prenatal examinations and is used to evaluate nutritional statuses during pregnancy [10]. The Institute of Medicine (IOM) developed GWG guidelines in 1990 and updated them in 2009 to guide clinical practice [11]. However, previous studies have used inconsistent definitions and have had different findings for GWG above typical guidelines as well as its associated GDM risk [12]. Currently, individuals may try to reduce GWG by improving the diet and exercise habits of women with obesity, with hopes of preventing GDM [13–18]. However, the relationship between pre-pregnancy body mass index (BMI), GWG, and GDM remains unclear [19]. Furthermore, differences in the risk of GDM in women carrying a male or female fetus exist [20, 21], though this association has not been adequately studied.

This study aimed to explore the influence of pre-pregnancy BMI and GWG on GDM as well as sex-specific effects that may exist.

## Methods

### Study design

This study established an observational cohort to monitor the weight of pregnant women from the beginning of pregnancy until the day of their GDM screening, typically between 24 and 28 gestational weeks. We analyzed the relationship between BMI and GWG as well as the risk of GDM. Potential confounding factors, including maternal age, multiparity, active smoking, education, and family history of diabetes, were adjusted for in the multivariable analysis.

### Population and data collection

**Fig. 1:**
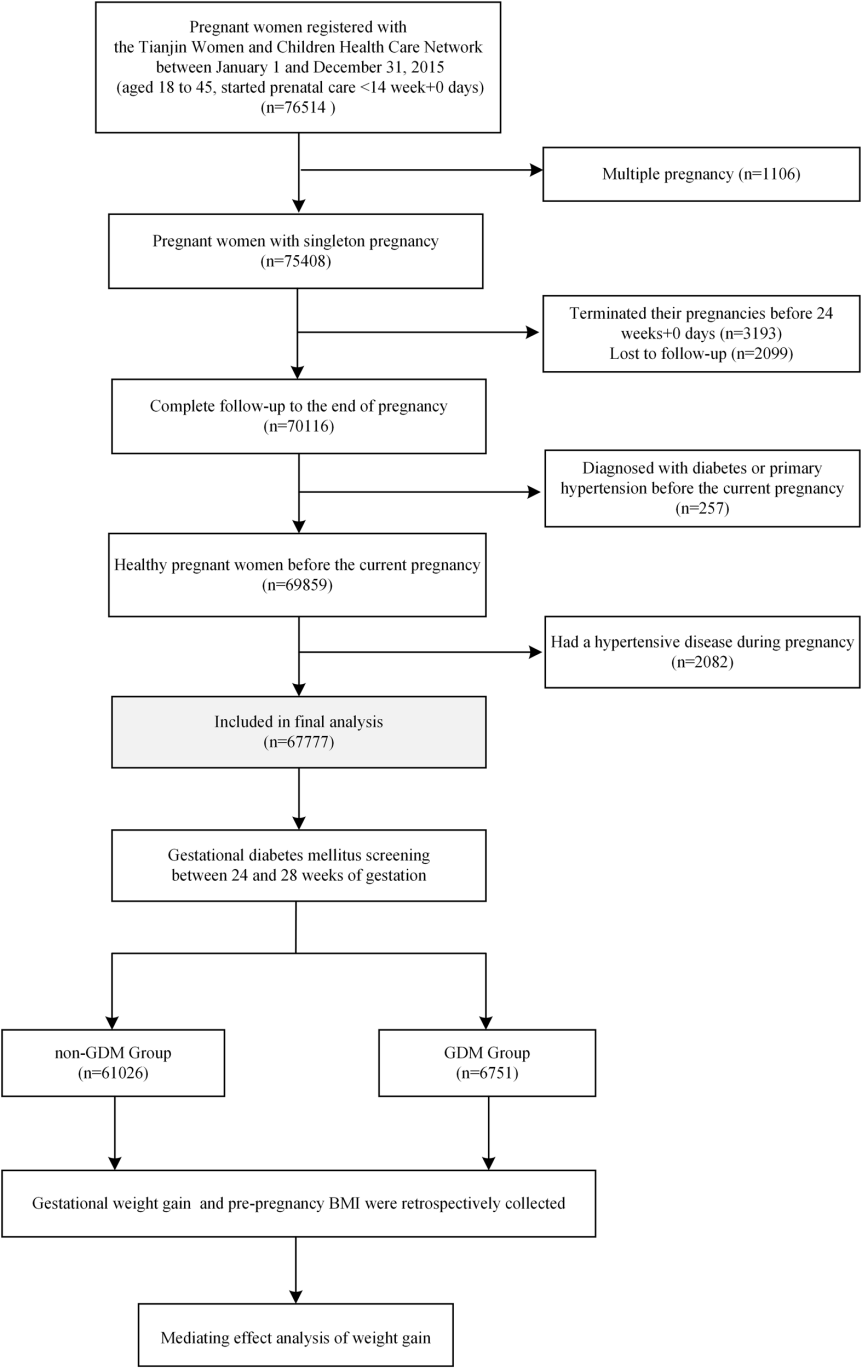
Study flow chart. GDM gestational diabetes mellitus.

This cohort included all singleton pregnant individuals aged 18–45 who were registered with the Tianjin Women and Child Health Care Network between January 1 and December 31, 2015. Pregnant women were excluded if they had: (1) been diagnosed with diabetes or primary hypertension before the current pregnancy, (2) had a hypertensive disease during pregnancy, (3) started prenatal care later than 13 weeks+6 days, or (4) terminated their pregnancies before 24 weeks+0 days (see Fig. 1).

Prenatal examination data were collected from the Tianjin Women and Children Health Care Network database, a government-administered public health system covering all communities in Tianjin, China and with antenatal care coverage rates of the local pregnant population exceeding 95%. At registration, each pregnant woman received a unique identification number that linked the antenatal care information recorded by different care providers. Basic information included date of birth, ethnicity, gravidity, parity, last menstrual period, history of chronic disease, family history of chronic and genetic diseases, and routine prenatal measurements and tests, such as height, weight, blood pressure, routine complete blood counts, urine test, blood glucose, liver, and kidney function at the first prenatal visit. Maternal weight, blood pressure, pregnancy complications, and medical treatments were continuously recorded. Antenatal care information was anonymized exported from the database, with only unique identification numbers used. The study was approved by the Ethics Committee for Clinical Research of Tianjin Women’s and Children’s Health Center. The need for written informed consent was waived due to this was retrospective analysis of data routinely collected from participants.

### Screening and diagnosis of gestational diabetes mellitus

All pregnant women were screened for diabetes between 24 and 28 weeks of gestation. After the publication of the 2010 International Association of Diabetes in Pregnancy Study Group (IADPSG) recommendations, our antenatal care system considered adopting the IADPSG criteria of interpreting the oral glucose tolerance test (OGTT) results. However, due to local conditions and an attempt at minimizing changes in public health management, the previous two-step testing method was maintained, which did not comply with the IADPSG one-step criteria [22]. In this study, a 50-g 1-h glucose-challenge test was used to screen pregnant women for diabetes at community hospitals. Women with plasma glucose (PG) ≥ 7.8 mmol/L were then referred to the Tianjin Women and Children’s Health Center for a 75 g OGTT. Participants were diagnosed with GDM and defined as the GDM group if their PG with a 75 g OGTT met one or more of the following criteria: (1) fasting PG ≥ 5.1 mmol/L, (2) 1-h PG ≥ 10.0 mmol/L, or (3) 2-h PG ≥ 8.5 mmol/L [1, 2]. Pregnant women whose PG did not reach these cutoff points were defined as the non-GDM group.

### Calculation of weight gain

GWG was defined as the difference between the final and baseline weights. Self-reported pre-pregnancy or measured weight in the first trimester is usually used to calculate pre-pregnancy BMI and GWG [11]. Although the accuracy of self-reported pre-pregnancy weight is uncertain, it can be easily collected or retrieved from medical records. In the present study, the mean differences between the two weight measurements were under 2 kg, which had little impact on BMI classification and GWG calculation [23]. Thus, it seemed to be a suitable choice to define the baseline weight. The final weight was measured on the day of diabetes screening tests that took place between 24 and 28 weeks of gestation. In addition, the gestational age of weight was adjusted for in the analysis.

BMI was calculated by dividing weight in kilograms by the square of height in meters. Participants were divided into four groups based on pre-pregnancy BMIs using the World Health Organization’s (WHO) BMI classification criteria: underweight (BMI < 18.5 kg/m^2^), normal weight (BMI 18.5–24.9 kg/m^2^), overweight (BMI 25.0–29.9 kg/m^2^), and obesity (BMI ≥ 30.0 kg/m^2^).

### Statistical analysis

IBM SPSS Statistics for Windows (Version 21.0. Armonk, NY: IBM Corp), R statistical software (R version 4.0.3, Comprehensive R Archive Network), and GraphPad Prism 8 (San Diego, CA: GraphPad Software) were used for data analysis and figure drawing. Normally distributed continuous variables were presented as means (standard deviations) and were compared between two groups using a *t*-test of independent samples. Moreover, non-normally distributed continuous variables were presented as medians (interquartile ranges), and an independent sample Mann–Whitney *U* test was performed to compare the two groups. Categorical variables were presented as frequencies (percentages) and were compared using the chi-square test. Restricted cubic spline (RCS) analysis was used in the logistic regression to assess nonlinear associations of BMI or weight gain with GDM, while linear regression analysis was performed to analyze the effects of pre-pregnancy BMI on GWG. Logistic regression analysis was performed to analyze the effects of BMI and GWG on GDM. A two-tailed *P* value of less than 0.05 was considered statistically significant, and multiple imputations were performed for missing values.

Furthermore, mediation analysis was conducted to determine whether GWG could mediate the relationship between pre-pregnancy BMI and GDM. Theoretically, if the independent variable X has a certain influence on the dependent variable Y through a certain variable M, then M plays a mediating role between variables X and Y and would therefore be the mediator. Mediation analysis can help explain mechanisms in the relationship between independent and dependent variables. In the most common mediation model, X, Y, and M are continuous variables. Using linear regressions, the mediation analysis equations were as follows:

1 $${{{\mathrm{Y}}}} = {{{\mathrm{cX}}}} + e_1$$

2 $${{{\mathrm{M}}}} = aX + e_2$$

3 $${{{\mathrm{Y}}}}^{\prime} = {{{\mathrm{c}}}}^{\prime} {{{\mathrm{X}}}} + {{{\mathrm{bM}}}} + e_3$$

*c* is the total effect of X on Y, *a × b* is the mediating effect through mediator M, and *c’* is the direct effect. When only one mediator exists, *c* = *c’*+*a × b*. The mediating effect was measured using the equation *c-c’* = *a × b*.

In this study, the dependent variable Y was GDM, the independent variable X was pre-pregnancy BMI, and the mediator M was GWG. Consequently, logistic regressions were used in place of the standard linear regression [24], where we applied the mediation model with X and M as continuous variables and Y as a binary variable with the following equations:

4 $${{{\mathrm{Y}}}}^{\prime} = i_4 + {{{\mathrm{cX}}}} + e_4$$

5 $${{{\mathrm{M}}}} = i_5 + aX + e_5$$

6 $$Y^{\prime\prime} = i_6 + {{{\mathrm{c}}}}^{\prime} {{{\mathrm{X}}}} + {{{\mathrm{bM}}}} + e_6$$

7 $$Y^{\prime} = LogitP\left( {Y = 1{{{\mathrm{|}}}}X} \right) = \ln \frac{{P\left( {Y = 1{{{\mathrm{|}}}}X} \right)}}{{P\left( {Y = 0{{{\mathrm{|}}}}X} \right)}}$$

8 $$Y^{\prime\prime} = LogitP\left( {Y = 1{{{\mathrm{|}}}}M,X} \right) = \ln \frac{{P\left( {Y = 1{{{\mathrm{|}}}}M,X} \right)}}{{P\left( {Y = 0{{{\mathrm{|}}}}M,X} \right)}}$$

As the dependent variable Y was binary, a logistic regression was adopted for Eqs. 4 and 6. The mediator M was continuous; therefore, linear regression was adopted in Eq. 5 [25, 26]. Here, the regression coefficient *a* came from the regression of the continuous variable M to X (the scale of the continuous variable), while regression coefficient *b* came from the regression of the binary dependent variable Y to M, X (the scale of logit). Therefore, the two regression coefficients were not on the same scale and were not comparable. For the regression coefficients to have the same scale, Lacobucci proposed the Sobel method [25]. A *t*-test was used to test the significance of regression coefficient *a* in the linear regression, and the tested statistic was *t* = *a*/SE(*a*). Typically, when sample sizes increase to more than 30 degrees of freedom, the *t*-test can be viewed as a *z*-test, which can be written as *Z*_*a*_ = *a*/SE (*a*). In the logistic regression, the significance of the regression coefficient *b* was tested using the Wald χ ^2^ test, and the test statistic was calculated as χ^2^ = *b*/SE(*b*)^2^. The square root of the test statistic is *b*/SE(*b*), which is the *t*-test statistic. When sample sizes increase to more than 30 degrees of freedom, *Z*_*b*_ = *b*/SE(*b*). After the regression coefficients *a* and *b* were converted into *Z*_*a*_ and *Z*_*b*_, they were on the same scale. Therefore, the size of the mediating effect of this model with binary dependent variables was *Z*_*a*_ *×* *Z*_*b*_, and a significance test of the mediating effect was used to test the significance of *Z*_*a*_ *×* *Z*_*b*_. The statistics were calculated as follows:

9 $$Z = \frac{{Z_{a \times b}}}{{{{{\mathrm{SE}}}}(Z_{a \times b})}} = \frac{{Z_a \times Z_b}}{{SE(Z_{a \times b})}} = \frac{{Z_a \times Z_b}}{{\sqrt {Z_a^2 + Z_b^2 + 1} }}$$

MacKinnon and Cox suggested applying the distribution-of-the-product method to build confidence intervals (CIs) for the mediating effect. The RMediation software package with R software was automatically operated to obtain the asymmetric CIs of *Z*_*a*_ *×* *Z*_*b*_ [27]. A significant mediating effect was defined as a CI that did not include zero (asymmetric interval).

## Results

### Population characteristics

A total of 67,777 pregnant women were included in this study (Fig. 1). Among them, 7055 (10.4%) were underweight, 45,880 (67.7%) were normal weight, 11,894 (17.5%) were overweight, and 2948 (4.3%) were obesity. Of the total participants, 6751 (10.0%) were diagnosed with GDM and defined as the GDM group, while 61,026 (90.0%) were defined as the non-GDM group. Compared to the non-GDM group, pregnant women in the GDM group were significantly older, had higher blood pressures (*P* < 0.001), had higher proportions of individuals with >12 years of education, higher levels of active smoking, family histories of diabetes, and higher proportions of carrying a male fetus (*P* < 0.05). Furthermore, these women had higher pre-pregnancy BMIs and lower levels of weight gain (*P* < 0.001) (Table 1).

**Table 1:** Characteristics of pregnant women in the GDM group and non-GDM group.

Factor	non-GDM group	GDM group	*t/χ*^*2*^	*P* value
*n* (%)	61026 (90.0%)	6751 (10.0%)		
Age, year	27.97 (4.10)	29.78 (4.18)	−33.929	<0.001
Ethnic Han, *n* (%)	58400 (95.7%)	6473 (95.9%)	0.508	0.476
Multiparity, *n* (%)	18182 (29.8%)	2156 (31.9%)	13.281	0.601
Education >12 years, *n* (%)	38075 (62.4%)	4474 (66.3%)	39.166	<0.001
Active smoking, *n* (%)	223 (0.4%)	47 (0.7%)	16.762	0.001
Family history of diabetes, *n* (%)	1199 (2.0%)	279 (4.1%)	133.936	<0.001
SBP, mmHg	106.44 (16.54)	109.57 (21.14)	−14.324	<0.001
DBP, mmHg	68.89 (7.78)	70.97 (8.30)	−19.662	<0.001
Gestational age of weight gain measure, week	25.95 (0.95)	25.97 (0.91)	−1.669	0.095
Pre-pregnancy BMI, kg/m^2^	22.37 (3.59)	24.29 (4.18)	−36.44	<0.001
Gestational weight gain, kg	7.77 (4.38)	7.11 (4.52)	−15.213	<0.001^a^
Fetal sex (boy), *n* (%)^b^	31373 (51.4%)	3561 (52.7%)	4.316	0.038

*GDM* gestational diabetes mellitus, *SBP* systolic blood pressure, *DBP* diastolic blood pressure, *BMI* body mass index.

^a^Independent sample Mann–Whitney *U* test was performed; ^b^sex determination was not performed in eight stillbirths.

### Relationship among pre-pregnancy body mass index, gestational weight gain, and gestational diabetes mellitus

#### Pre-pregnancy body mass index and gestational diabetes mellitus

In this study, RCS analysis was performed to verify the relationship between pre-pregnancy BMI and GDM. In general, the risk of GDM nonlinearity increased with an increase in BMI (nonlinear *P* < 0.001) (Supplementary Fig. 1A). However, subgroup analysis showed a linear relationship between BMI and GDM in each BMI category (all nonlinear *P* > 0.05) (Supplementary Fig. 1B). Logistic regression analysis confirmed that BMI significantly increased the risk of GDM when maternal BMI was ≥18.5 kg/m^2^ (BMI 18.5–24.9 kg/m^2^, *OR* 1.175, 95% C*I* 1.153–1.198, *P* < 0.001; BMI 25.0–29.9 kg/m^2^, *OR* 1.110, 95% CI 1.072–1.150, *P* < 0.001; BMI ≥ 30.0 kg/m^2^, *OR* 1.075, 95% CI 1.039–1.112, *P* < 0.001) (Supplementary Table 1).

#### Gestational weight gain and gestational diabetes mellitus

**Fig. 2:**
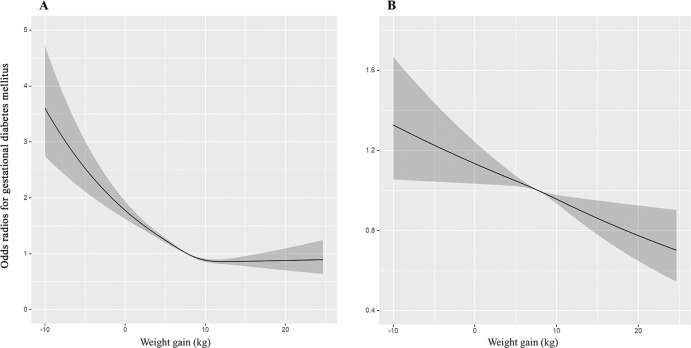
RCS curve of weight gain to odds radios for gestational diabetes mellitus. **A** Univariate regression analysis. **B** Multivariable regression analysis, adjusting for maternal age and pre-pregnancy body mass index. Odds ratios are indicated by solid lines and 95% confidence intervals are indicated by shaded areas. The reference point is the lowest value for gestational diabetes mellitus, with five knots placed at the 5th, 27.5th, 50th, 72.5th, and 95th percentiles of weight gain distribution. *RCS* restricted cubic spline.

Logistic regression analysis showed that weight gain negatively contributed to GDM after adjusting for pre-pregnancy BMI and maternal age (*OR* 0.945, 95% CI 0.938–0.952, *P* < 0.001; *AOR* 0.983, 95% CI 0.976–0.991, *P* < 0.001) (Supplementary Table 2). The RCS curve of GWG and OR for GDM was inverse J-shaped (nonlinear *P* < 0.001) (Fig. 2A). However, after adjusting for pre-pregnancy BMI, GWG was negatively correlated with GDM (nonlinear *P* = 0.680) (Fig. 2B). Moreover, subgroup analysis showed a linear relationship between GWG and GDM in each BMI category (all nonlinear *P* > 0.05) (Supplementary Fig. 2). We further verified by logistic regression analysis that GWG had a significant negative effect on GDM when pre-pregnancy BMI was 18.5–24.9 kg/m^2^ (*AOR* 0.984, 95% CI 0.974–0.994, *P* = 0.002), and 25.0–29.9 kg/m^2^ (*AOR* 0.980, 95% CI 0.967–0.994, *P* = 0.004) (Supplementary Table 3).

#### Body mass index and gestational weight gain

**Fig. 3:**
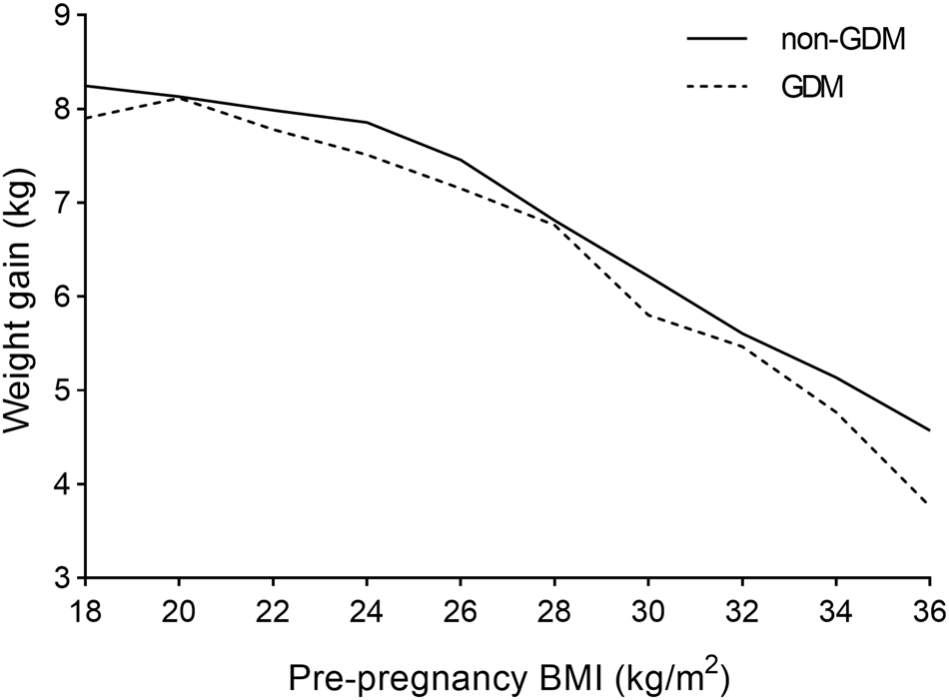
Correlation between pre-pregnancy BMI and weight gain in the GDM and non-GDM group. The pre-pregnancy BMI categories are 2 kg/m^2^ each, and weight gain is a continuous variable. The solid line represents the non-GDM group and the dotted line represents the GDM group. GDM gestational diabetes mellitus, BMI body mass index.

Our previously noted results showed that pre-pregnancy BMI and GWG appeared to have opposite effects on GDM. We also investigated the influence of pre-pregnancy BMI on weight gain. GWG decreased with an increase in pre-pregnancy BMI in both the GDM and non-GDM groups (Fig. 3). GWG in the GDM group was lower than that in the non-GDM group. Therefore, we formulated a linear regression model with GWG as the dependent variable and pre-pregnancy BMI as the independent variable. Results showed that pre-pregnancy BMI negatively contributed to GWG (*β* = −0.239, 95% CI = −0.246 to −0.233, *P* < 0.001), which remained true after adjusting for confounding factors (*β* = −0.232, 95% CI = −0.239 to −0.225, *P* < 0.001) (Table 2).

**Table 2:** Linear regression analysis of the effect of pre-pregnancy BMI on weight gain.

Group	*n*	*β*	95% CI of *β*	SE	*t*	*P* value
		Model 1				
All BMI	67,777	−0.239	−0.246 to −0.233	0.004	−68.010	<0.001
BMI category (kg/m^2^)						
<18.5	7055	−0.186	−0.272 to −0.099	0.044	−4.202	<0.001
18.5–24.9	45,880	−0.168	−0.185 to −0.151	0.009	−19.049	<0.001
25.0–29.9	11,894	−0.353	−0.401 to −0.305	0.025	−14.346	<0.001
≥30.0	2948	−0.217	−0.278 to −0.155	0.031	−6.924	<0.001
		Model 2				
All BMI	67,777	−0.232	−0.239 to −0.225	0.004	−64.554	<0.001
BMI category (kg/m^2^)						
<18.5	7055	−0.182	−0.269 to −0.095	0.044	−4.102	<0.001
18.5–24.9	45,880	−0.160	−0.177 to −0.142	0.009	−17.947	<0.001
25.0–29.9	11,894	−0.344	−0.392 to −0.296	0.025	−14.001	<0.001
≥30.0	2948	−0.208	−0.270 to −0.146	0.031	−6.623	<0.001

*BMI* body mass index, *CI* confidence interval, *SE* standard error.

Model 1: Univariate regression analysis, with gestational weight gain as the dependent variable.

Model 2: Multivariable regression analysis, adjusting for maternal age, parity, education level, active smoking, and family history of diabetes.

### Mediating effect analysis

Pre-pregnancy BMI, GWG, and GDM are known to be interrelated. Therefore, we explored whether GWG played a mediating role in the association between BMI and GDM. Table 3 shows that GWG had a significant mediating effect on BMI and GDM (*Z*_*a*_ *×* *Z*_*b*_ CIs 0.00243–0.00618) (Supplementary Fig. 3). In the analysis of different BMI categories, no significant mediating effects were noted in the BMI < 18.5 kg/m^2^ or BMI ≥ 30.0 kg/m^2^ groups. In addition, the effect size of the BMI 18.5–24.9 kg/m^2^ category was greater than that in the BMI 25.0–29.9 kg/m^2^ category (*Z*_*a*_ *×* *Z*_*b*_ 67.20 vs. 48.41).

**Table 3:** Mediation analysis of weight gain on the relationship between pre-pregnancy BMI and GDM.

Group		*n*	*a*	SE*(a)*	*Z*_*a*_	*b*	SE*(b)*	*Z*_*b*_	*Z*_*a*_ *× Z*_*b*_	CI *of Z*_*a*_ *× Z*_*b*_		
Regardless of fetal sex												
BMI (kg/m^2^)	All	67777	−0.239	0.004	−59.75	−0.018	0.004	−4.50	268.88	0.00243	to	0.00618^*^
	<18.5	7055	−0.186	0.044	−4.23	−0.004	0.018	−0.22	0.94	−0.00607	to	0.00777
	18.5–24.9	45880	−0.168	0.009	−18.67	−0.018	0.005	−3.60	67.20	0.00137	to	0.00473^*^
	25.0–29.9	11894	−0.353	0.025	−14.12	−0.024	0.007	−3.43	48.41	0.00358	to	0.01359^*^
	≥30.0	2948	−0.217	0.031	−7.00	−0.019	0.011	−1.73	12.09	−0.00055	to	0.00924
Carrying a male fetus												
BMI (kg/m^2^)	All	34934	−0.230	0.005	−46.00	−0.018	0.005	−3.60	165.60	0.00188	to	0.00641^*^
	<18.5	3546	−0.149	0.063	−2.37	0.010	0.025	0.40	−0.95	−0.01048	to	0.00644
	18.5–24.9	23720	−0.162	0.012	−13.50	−0.021	0.007	−3.00	40.50	0.00117	to	0.00574^*^
	25.0–29.9	6183	−0.329	0.034	−9.68	−0.017	0.010	−1.70	16.45	−0.00085	to	0.01236
	≥30.0	1485	−0.170	0.042	−4.05	−0.020	0.016	−1.25	5.06	−0.00190	to	0.00970
Carrying a female fetus												
BMI (kg/m^2^)	All	32835	−0.249	0.005	−49.80	−0.019	0.005	−3.80	189.24	0.00229	to	0.00718^*^
	<18.5	3509	−0.223	0.062	−3.60	−0.022	0.028	−0.79	2.83	−0.00749	to	0.01910
	18.5–24.9	22154	−0.175	0.013	−13.46	−0.014	0.007	−2.00	26.92	0.00005	to	0.00493^*^
	25.0–29.9	5710	−0.378	0.036	−10.50	−0.031	0.010	−3.10	32.55	0.00422	to	0.01977^*^
	≥30.0	1462	−0.270	0.046	−5.87	−0.018	0.015	−1.20	7.04	−0.00306	to	0.01352

*BMI* body mass index, *GDM* gestational diabetes mellitus, *CI* confidence interval, *SE* standard error.

Sex determination was not performed in eight stillbirths. *a* = raw (unstandardized) regression coefficient for the association between BMI (X) and weight gain (M, mediator); *b* = raw coefficient for the association between weight gain (M, mediator) and GDM (Y) (X is also a predictor of Y). SE(*a*) = standard error of *a*; SE(*b*) = standard error of *b*. *Z*_*a*_ = *a*/SE(*a*), *Z*_*b*_ = *b*/SE(*b*). We used *Z*_*a*_ × *Z*_*b*_ to measure the size of the mediating effect. ^*^Statistical significance of the mediating effect was defined as a CI that did not include zero.

### Fetal sex specificity

As shown in Table 1, differences in the distribution of fetal sex between the GDM and non-GDM groups were noted. Logistic analysis showed that, compared with carrying a female fetus, carrying a male fetus was an independent risk factor for the development of GDM (*OR* 1.055, 95% CI 1.003–1.109, *P* = 0.038). In addition, in comparing weight gain, we found that mothers carrying female fetuses gained less weight than those carrying male fetuses, especially if pre-pregnancy BMI was greater than 25.0 kg/m^2^ (Supplementary Table 4). Subgroup analysis showed that this difference was mainly observed in the non-GDM group and was not significant in the GDM group (Supplemental Table 4).

A mediating effect was also observed in the subgroup analysis of fetal sex. Table 3 shows that the effect size in women carrying a female fetus was greater than that in women carrying a male fetus (*Z*_*a*_ *×* *Z*_*b*_ 189.24 vs. 165.60). Notably, in the BMI 25.0–29.9 kg/m^2^ category, the mediating effect was only observed in women carrying a female fetus (*Z*_*a*_ *× Z*_*b*_ CIs 0.00422 to 0.01977) and not in those carrying a male fetus (*Z*_*a*_ *× Z*_*b*_ CIs −0.00085 to 0.01236). In contrast, in the BMI 18.5–24.9 kg/m^2^ category, the effect size in women with male fetuses was greater than that in those with female fetuses (*Z*_*a*_ *× Z*_*b*_ 40.50 vs. 26.92). Our results indicated that the mediating effect of weight gain during pregnancy was sex-specific.

## Discussion

Weight gain during pregnancy is considered an indicator of the accumulation of maternal fat, expansion of body fluids, and growth of the breast, uterus, placenta, and fetus. It is closely associated with nutritional intake, physical exercise, and other metabolic conditions during pregnancy [28], and is therefore regarded as an important health monitoring item in pregnancy care [29]. Managing weight gain may prevent GDM in pregnant women with obesity [30, 31]. Weight gain is also widely used to evaluate the effects of lifestyle interventions [32–35]. However, the role of weight gain in GDM has not been fully understood [19].

In this study, RCS analysis showed a negative correlation between weight gain and the risk of GDM. Additionally, we confirmed that pre-pregnancy BMI can increase the risk of GDM, which is consistent with previous studies [22]. Therefore, we focused on the influence of pre-pregnancy BMI on GWG. Linear regression models demonstrated that weight gain decreased significantly as pre-pregnancy BMI increased. Our findings demonstrated a complex relationship between BMI, GWG, and GDM.

Mediation analysis confirmed a significant mediating effect of GWG on pre-pregnancy BMI and GDM. Furthermore, subgroup analysis revealed that this mediating effect existed only in normal weight and overweight pregnant women, with the effect size being greater in the normal weight group. This may be due to weight gain decreases with higher pre-pregnancy BMI. Normal weight women may have gained more weight than those that were overweight, making the mediating effect more obvious.

Furthermore, we confirmed that differences existed in fetal sex subgroup analysis. Weight gain in women carrying female fetuses showed a more powerful mediating effect than that in women carrying male fetuses in the overweight group. This phenomenon may follow sex-specific programming theory. Based on the fetal origin hypothesis, female fetuses make more adaptive changes in utero than male fetuses in response to maternal metabolic disturbance [36]. The intrauterine environment of overweight pregnant women is characterized by overnutrition, and female fetuses may actively reduce their weight gain, which may help maintain maternal and metabolic homeostasis.

These results suggest that weight gain may be a good predictor of GDM and can be used to evaluate the effectiveness of preventive measures in normal weight and overweight women. In addition, weight evaluation may be more effective for women carrying female fetuses than for those carrying male fetuses.

### Strengths and limitations

This study was based on a government-administered health care system that covered all community populations in Tianjin, China. Data acquisition and quality control were monitored at multiple levels, which made the data representative and objective. In addition, a sufficient number of underweight women were included in this study. Therefore, the results could be a meaningful supplement to the IOM guidelines. BMI was categorized according to the WHO classification, which may make the conclusions applicable to and a reference for populations outside of Asia. However, the potential weaknesses of this study are worth considering. All pregnant women were from China, and the sample size of the obesity group was relatively small, which may have resulted in insignificant mediating effects in women with obesity.

## Supplementary information

Supplemental Material

## Data Availability

The datasets analyzed during this study are available from the corresponding author on reasonable request.
