# Supplementary material for: Sex-specific mediating effect of gestational weight gain between pre-pregnancy body mass index and gestational diabetes mellitus
Source: Nutr Diabetes. 2022 Apr 25;12:25. doi: 10.1038/s41387-022-00203-5 (PMC9039078; doi:10.1038/s41387-022-00203-5)
Supplement: Supplementary file 1 — Supplemental Material [file 41387_2022_203_MOESM1_ESM.docx]

**Supplemental Material**


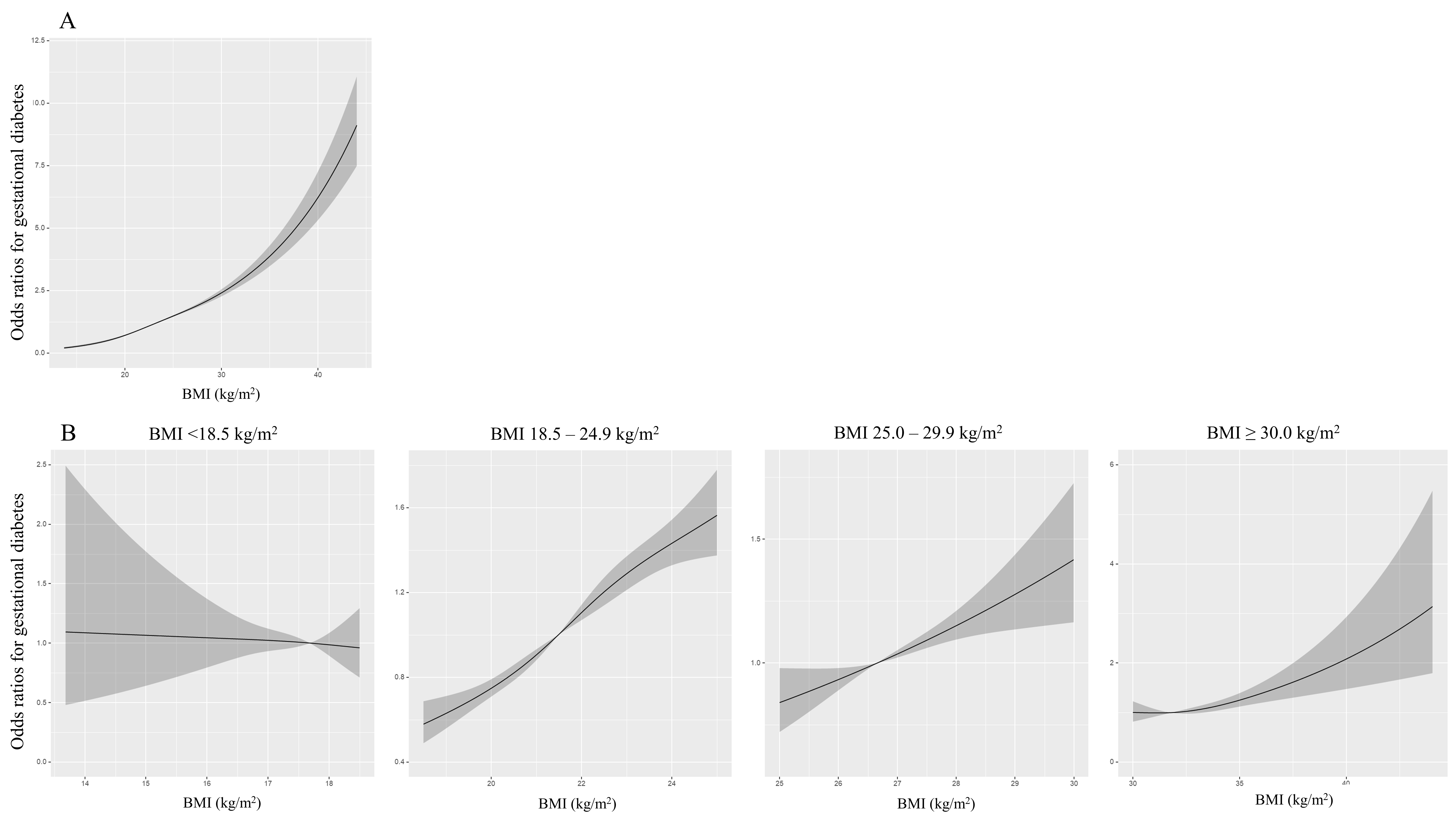


**Supplementary Figure 1** RCS curve of pre-pregnancy BMI to odds radios for gestational diabetes

(A) Samples taken from pregnant women with all BMI (n=67777); (B) Samples taken from pregnant women with four BMI categories.

Odds ratios are indicated by solid lines and 95% confidence intervals are indicated by shaded areas. The reference point is the lowest value for gestational diabetes, with four knots placed at the 5th, 35th, 65th, and 95th percentiles of weight gain distribution. Abbreviations: RCS = restricted cubic spline, BMI = body mass index.


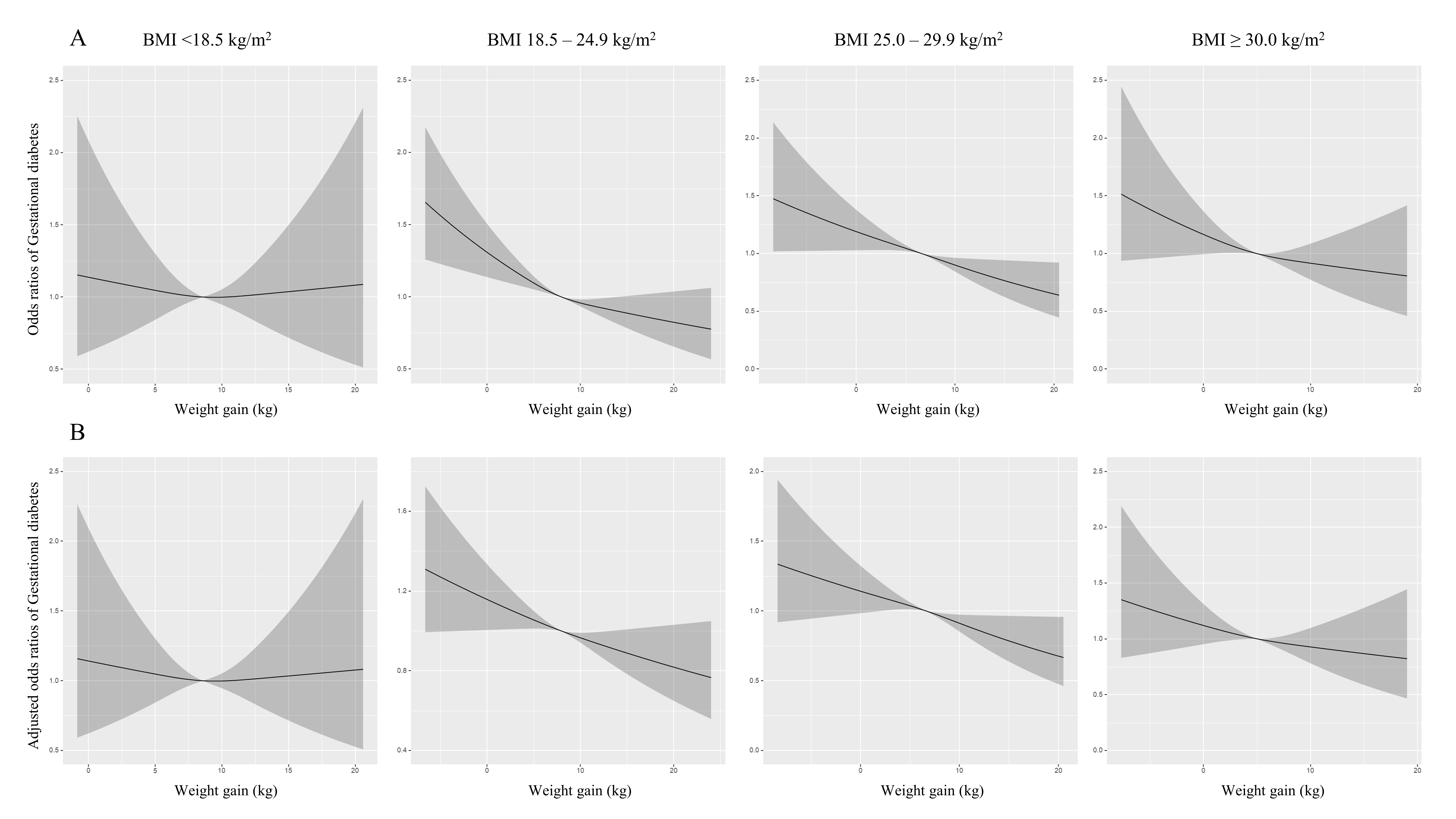


**Supplementary Figure 2** RCS curve of weight gain to odds radios for gestational diabetes in four BMI categories

(A) Univariate regression analysis; (B) Multivariable regression analysis, adjusting for maternal age and pre-pregnancy BMI.

Odds ratios are indicated by solid lines and 95% confidence intervals are indicated by shaded areas. The reference point is the lowest value for gestational diabetes, with five knots placed at the 5th, 27.5th, 50th, 72.5th, and 95th percentiles of weight gain distribution. Abbreviations: RCS = restricted cubic spline, BMI = body mass index.


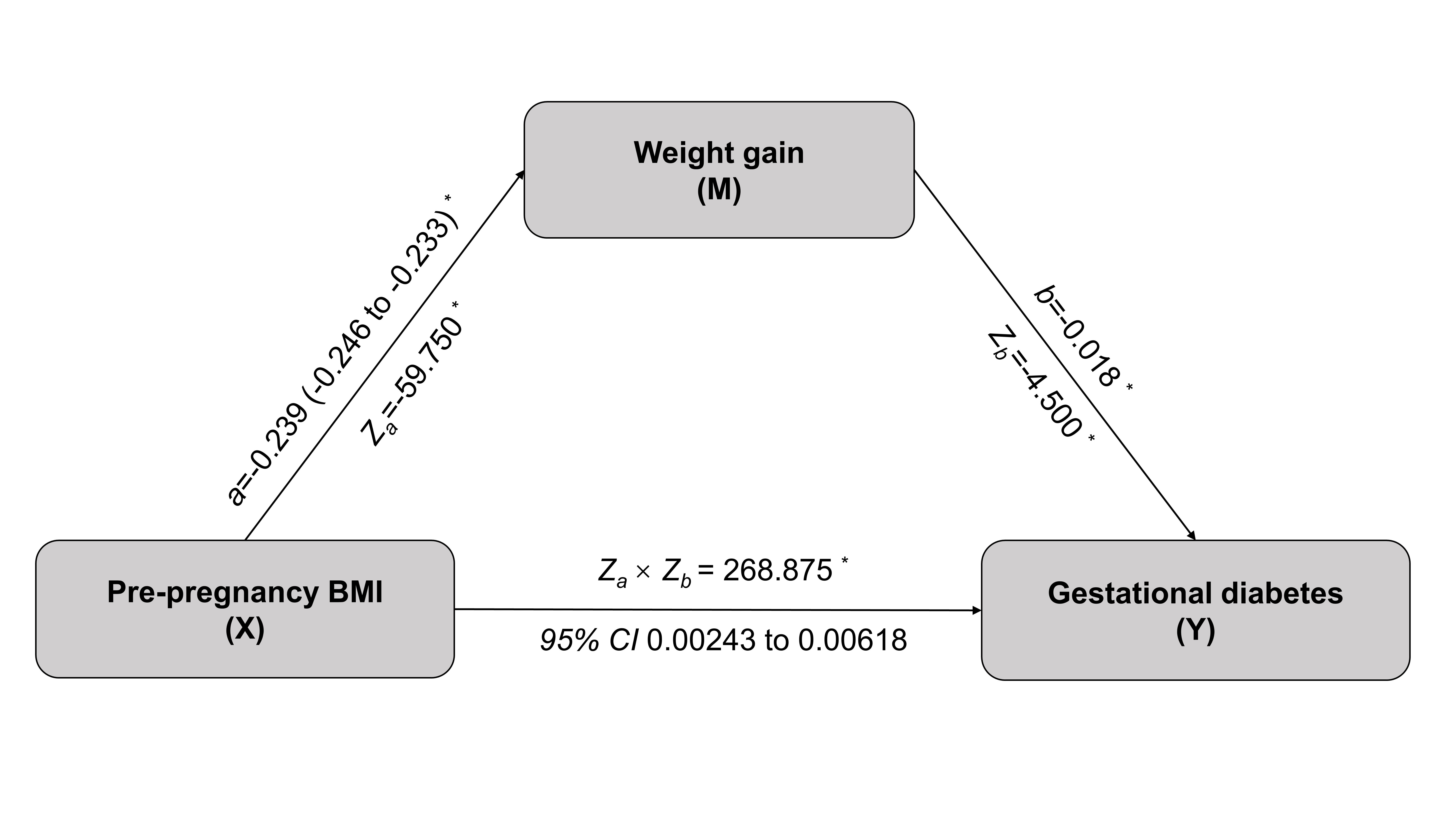


**Supplementary Figure 3** Mediation analysis of weight gain on the relationship between pre-pregnancy BMI and gestational diabetes

*a*: effect of prepregnancy BMI on weight gain; *b*: effect of weight gain on gestational diabetes; *Z_a_*×*Z_b_*: used to measure the size of the mediating effect of weight gain on gestational diabetes. *a* = raw (unstandardized) regression coefficient for the association between BMI (X) and weight gain (M, mediator); *b* = raw coefficient for the association between weight gain (M, mediator) and gestational diabetes (Y) (X is also a predictor of Y). SE(*a*) = standard error of *a*; SE(*b*) = standard error of *b*. *Z_a_* × *Z_b_* is used to measure the size of the mediating effect. **P*<0.05. The statistical significance of the mediating effect was defined as a *CI* that did not include zero. Abbreviations: BMI = body mass index, CI = confidence interval, SE = standard error.

**Supplementary Table 1** Logistic regression analysis of the effect of pre-pregnancy BMI on GDM

| Pre-pregnancy BMI (kg/m^2^) | *n* | *β* | *OR* | *95% CI of OR* | *P*-value |
| --- | --- | --- | --- | --- | --- |
| All BMI | 67777 | 0.122 | 1.130 | 1.123 - 1.137 | <0.001 |
| <18.5 | 7055 | -0.031 | 0.969 | 0.850 - 1.105 | 0.639 |
| 18.5 - 24.9 | 45880 | 0.161 | 1.175 | 1.153 - 1.198 | <0.001 |
| 25.0 - 29.9 | 11894 | 0.105 | 1.110 | 1.072 - 1.150 | <0.001 |
| ≥30.0 | 2948 | 0.072 | 1.075 | 1.039 - 1.112 | <0.001 |

Abbreviations: BMI = body mass index, GDM = gestational diabetes mellitus, OR = odds ratio, CI = confidence interval.

**Supplementary Table 2** Logistic regression analysis of the effect of weight gain on GDM in 67777 women

| Independent variable | *β* | *OR* | *95% CI of OR* | *P*-value |
| --- | --- | --- | --- | --- |
| Model 1 |  |  |  |  |
| Weight gain | -0.057 | 0.945 | 0.938 - 0.952 | <0.001 |
| Model 2 |  |  |  |  |
| Weight gain | -0.018 | 0.982 | 0.975 - 0.989 | <0.001 |
| Pre-pregnancy BMI | 0.118 | 1.125 | 1.118 - 1.132 | <0.001 |
| Model 3 |  |  |  |  |
| Weight gain | -0.017 | 0.983 | 0.976 - 0.991 | <0.001 |
| Pre-pregnancy BMI | 0.11 | 1.117 | 1.109 - 1.124 | <0.001 |
| Maternal age | 0.091 | 1.095 | 1.088 - 1.101 | <0.001 |

Abbreviations: GDM = gestational diabetes mellitus, BMI = body mass index, OR = odds ratio, CI = confidence interval.

**Supplementary Table 3** Logistic regression analysis of the effect of weight gain on GDM in different BMI categories

| BMI category (kg/m^2^) | *n* | *β* | *OR* | *95% CI of OR* | *P*-value |
| --- | --- | --- | --- | --- | --- |
|  |  | Model 1 |  |  |  |
| <18.5 | 7055 | -0.003 | 0.997 | 0.962 - 1.033 | 0.864 |
| 18.5 - 24.9 | 45880 | -0.025 | 0.975 | 0.965 - 0.985 | <0.001 |
| 25.0 - 29.9 | 11894 | -0.029 | 0.971 | 0.959 - 0.984 | <0.001 |
| ≥30.0 | 2948 | -0.024 | 0.976 | 0.955 - 0.997 | 0.025 |
|  |  | Model 2 |  |  |  |
| <18.5 | 7055 | -0.002 | 0.998 | 0.962 - 1.035 | 0.912 |
| 18.5 - 24.9 | 45880 | -0.016 | 0.984 | 0.974 - 0.994 | 0.002 |
| 25.0 - 29.9 | 11894 | -0.020 | 0.980 | 0.967 - 0.994 | 0.004 |
| ≥30.0 | 2948 | -0.020 | 0.980 | 0.959 - 1.002 | 0.076 |

Model1: Univariate regression analysis, with gestational weight gain as the dependent variable.

Model2: Multivariable regression analysis, adjusting for maternal age and pre-pregnancy BMI.

Abbreviations: GDM = gestational diabetes mellitus, BMI = body mass index, OR = odds ratio, CI = confidence interval.

**Supplementary Table 4** Compare the weight gain of pregnant women carrying a male and female fetus

| Group | BMI (kg/m^2^) | *n* | carrying a male fetus | *n* | carrying a female fetus | *P*-value^*^ |
| --- | --- | --- | --- | --- | --- | --- |
| all women | All BMI | 34934 | 7.76 (5.58 - 10.00) | 32835 | 7.66 (5.50 - 9.89) | 0.001 |
|  | <18.5 | 3546 | 8.66 (6.76 - 10.66) | 3509 | 8.50 (6.72 - 10.68) | 0.282 |
|  | 18.5 - 24.9 | 23720 | 8.00 (5.98 - 10.13) | 22154 | 7.92 (5.89 - 10.02) | 0.060 |
|  | 25.0 - 29.9 | 6183 | 6.73 (4.41 - 9.06) | 5710 | 6.52 (4.19 - 8.99) | 0.039 |
|  | ≥30.0 | 1485 | 5.06 (2.70 - 7.58) | 1462 | 4.87 (2.12 - 7.39) | 0.032 |
| non-GDM | All BMI | 31373 | 7.83 (5.67 - 10.00) | 29645 | 7.73 (5.58 - 9.96) | 0.001 |
|  | <18.5 | 3371 | 8.64 (6.77 - 10.65) | 3364 | 8.51 (6.73 - 10.68) | 0.362 |
|  | 18.5 - 24.9 | 21656 | 8.00 (6.00 - 10.16) | 20362 | 7.94 (5.92 - 10.05) | 0.003 |
|  | 25.0 - 29.9 | 5118 | 6.79 (4.44 - 9.13) | 4788 | 6.59 (4.29 - 9.00) | 0.106 |
|  | ≥30.0 | 1158 | 5.12 (2.84 - 7.50) | 1131 | 4.92 (2.16 - 7.42) | 0.040 |
| GDM | All BMI | 3561 | 7.17 (4.97 - 9.46) | 3190 | 7.04 (4.82 - 9.33) | 0.980 |
|  | <18.5 | 175 | 8.86 (6.75 - 10.91) | 145 | 8.31 (6.58 - 10.89) | 0.395 |
|  | 18.5 - 24.9 | 2064 | 7.61 (5.64 - 9.92) | 1792 | 7.68 (5.61 - 9.75) | 0.900 |
|  | 25.0 - 29.9 | 995 | 6.35 (4.19 - 8.62) | 922 | 6.11 (3.77 - 8.60) | 0.124 |
|  | ≥30.0 | 327 | 4.71 (1.96 - 7.81) | 331 | 4.65 (2.00 - 7.24) | 0.512 |

^*^ Independent sample Mann-Whitney U test was performed. Abbreviations: BMI = body mass index, GDM = gestational diabetes mellitus.
